# Dapagliflozin-Induced Erythrocytosis in Chronic Kidney Disease: A Rare Occurrence

**DOI:** 10.7759/cureus.58823

**Published:** 2024-04-23

**Authors:** Amit Pasari, Manish Balwani, Charulata Bawankule, Priyanka Tolani, Prasad Gurjar, Kapil Sejpal, Sunny Malde, Sushrut Gupta, Shubham Dubey, Pranjal Kashiv, Amol Bhawane

**Affiliations:** 1 Department of Nephrology, Jawaharlal Nehru Medical College, Datta Meghe Institute of Higher Education and Research, Wardha, IND; 2 Department of Nephrology, Saraswati Kidney Care Center, Nagpur, IND; 3 Department of Internal Medicine, Jawaharlal Nehru Medical College, Datta Meghe Institute of Higher Education and Research, Wardha, IND; 4 Department of Nephrology, All India Institute of Medical Sciences, Nagpur, IND

**Keywords:** hemoglobin, chronic kidney disease, erythrocytosis, dapagliflozin, sglt2 inhibitors

## Abstract

Erythrocytosis, a rare adverse effect associated with sodium-glucose cotransporter 2 inhibitors (SGLT2i), has been reported in diabetic patients, but its occurrence in those with chronic kidney disease (CKD) remains underrecognized. Here, we present two cases of dapagliflozin-related erythrocytosis in diabetic patients with CKD, highlighting the need for increased awareness among clinicians. Despite the established efficacy of SGLT2i in managing type 2 diabetes mellitus (T2DM) and its cardiovascular benefits, erythrocytosis poses a potential complication, necessitating thorough understanding and monitoring. While the precise mechanism of SGLT2i-induced erythrocytosis remains unclear, hypotheses include hemoconcentration and modulation of iron metabolism. Notably, our cases demonstrate a rapid onset of erythrocytosis, possibly exacerbated by CKD, emphasizing the importance of vigilant hemoglobin monitoring, especially in CKD patients on SGLT2i therapy. Timely discontinuation of dapagliflozin resulted in a significant reduction in hemoglobin levels, underscoring the critical role of early intervention in preventing erythrocytosis-related complications. This report advocates for routine hematological evaluation in CKD patients treated with SGLT2i to promptly detect and manage erythrocytosis, enhancing patient safety and improving clinical outcomes.

## Introduction

Sodium-glucose cotransporter 2 inhibitors (SGLT2i) have proven to be efficacious in the treatment of hyperglycemia and, due to their cardiovascular and renal benefits, have gained prominence in the management of type 2 diabetes mellitus (T2DM) [1]. In addition to their excellent efficacy, SGLT2i can have certain adverse effects. Erythrocytosis as a side effect of SGLT2i has been reported to range from 2% to 19% [2]. Although the exact mechanism remains unclear, the putative mechanism includes hemoconcentration, modulation of iron metabolism, and erythrocytosis stimulation by suppression of hypoxia-inducible factor (HIF)-1α and activation of HIF-2α [3]. There have been no reports of erythrocytosis related to SGLT2i in patients with chronic kidney disease (CKD). Here, we report two cases of patients with T2DM and CKD who developed erythrocytosis with the use of dapagliflozin.

## Case presentation

Case 1

**Figure 1:**
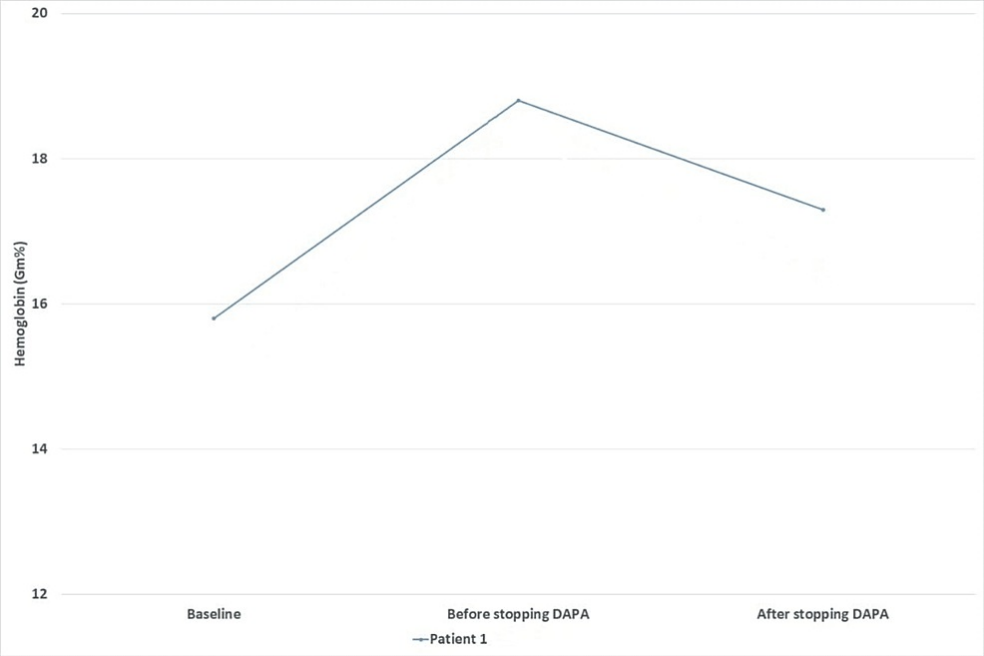
Changes in hemoglobin levels at different time points DAPA: dapagliflozin

A 70-year-old male with T2DM and chronic hypertension had comorbid non-proliferative diabetic retinopathy and underlying diabetic nephropathy. The patient was initiated on dapagliflozin (10 mg/day) for the management of hyperglycemia and renal dysfunction. Baseline investigations revealed a hemoglobin (Hb) level of 15.8 g/dL, red cell count of 4.62 million cells/mm^3^, serum creatinine of 2.40 mg/dL, and an estimated glomerular filtration rate (eGFR) of 28 mL/min/1.73 m^2^. Routine investigations, after seven months of continued use of dapagliflozin, detected erythrocytosis. The Hb raised to 18.8 g/dL, and the red cell count was 5.45 million cells/mm^3^. On a detailed evaluation to look for potential risk factors for erythrocytosis (e.g., smoking, splenomegaly, obstructive airway diseases, high-altitude residence, associated systemic symptoms, and other drugs stimulating erythropoiesis), we did not find any risk factors in a given case. Renal Doppler examination did not reveal any evidence of renal artery stenosis. In view of the erythrocytosis, dapagliflozin was immediately stopped. After a month of discontinuation, his Hb reduced to 17.3 g/dL and his red cell count to 5.08 million cells/mm^3^. His renal function had improved at this stage, with a serum creatinine of 1.92 mg/dL and an eGFR of 37 mL/min/1.73 m^2 ^(Table 1) (Figure 1).

**Table 1:** Summary of progression of laboratory parameters

Parameter	Baseline Value	Value After Seven Months of Dapagliflozin	Value After One Month of Dapagliflozin Discontinuation	Reference Range
Hemoglobin (Hb)	15.8 g/dL	18.8 g/dL	17.3 g/dL	13-16 g/dL
Red blood cell count	4.62 million cells/mm^3^	5.45 million cells/mm^3^	5.08 million cells/mm^3^	4.1-4.7 million cells/mm^3^
Serum creatinine	2.40 mg/dL	-	1.92 mg/dL	0.4-1.4 mg/dL
Estimated glomerular filtration rate (eGFR)	28 mL/min/1.73 m^2^	-	37 mL/min/1.73 m^2^	>60 mL/min/1.73 m^2^

Case 2

**Figure 2:**
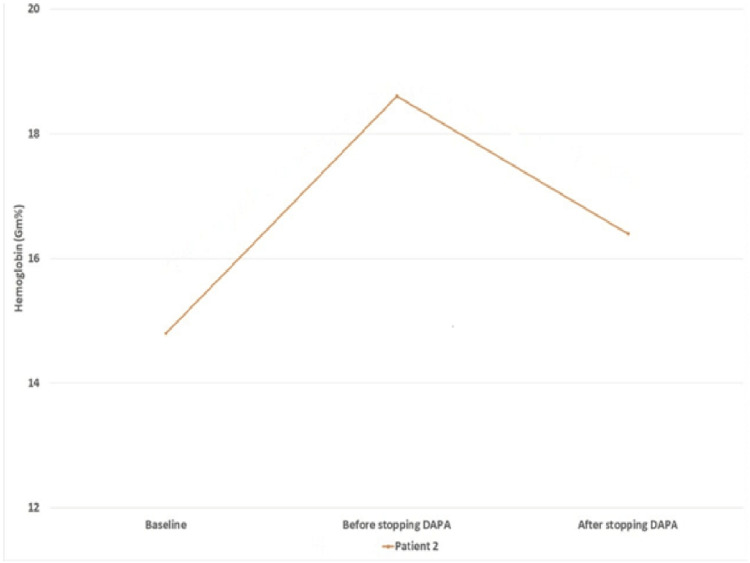
Changes in hemoglobin levels at different time points DAPA: dapagliflozin

This case is of a 64-year-old diabetic hypertensive male who had suspected non-diabetic CKD. He was initiated with dapagliflozin (5 mg/day) for the control of his hyperglycemia, which was uncontrolled despite three oral antihyperglycemic agents. His serum creatinine and eGFR before starting dapagliflozin were 2.3 mg/dL and 29 mL/min/1.73 m^2^, respectively. Baseline hematological investigations included Hb of 14.8 g/dL, red cell count of 4.44 million cells/mm^3^, and hematocrit of 39%. After six months of continued use of dapagliflozin, we observed changes in the hematological parameters. His Hb, red blood cell count, and hematocrit increased to 18.6 g/dL, 6.8 million cells/mm^3^, and 56%, respectively. In the absence of any other erythrocytosis-inducing drugs and no renal artery stenosis in renal Doppler, it was suggested that it could be related to dapagliflozin. After two months of discontinuing dapagliflozin, his Hb was reduced to 16.4 g/dL, red blood cell counts to 5.6 million cells/mm^3^, and hematocrit to 48%. At the time of discontinuation of dapagliflozin, his serum creatinine and eGFR were 2.21 mg/dL and 30 mL/min/1.73 m^2^, which changed to 2.48 mg/dL and 26 mL/min/1.73 m^2^ after two months of dapagliflozin discontinuation (Table 2) (Figure 2).

**Table 2:** Summary of progression of laboratory parameters

Parameter	Baseline Value	Value After Six Months of Dapagliflozin	Value After Two Months of Dapagliflozin Discontinuation	Reference Range
Hemoglobin (Hb)	14.8 g/dL	18.6 g/dL	16.4 g/dL	13-16 g/dL
Red blood cell count	4.44 million cells/mm^3^	6.8 million cells/mm^3^	5.6 million cells/mm^3^	4.1-4.7 million cells/mm^3^
Hematocrit	39%	56%	48%	37%-47%
Serum creatinine	2.3 mg/dL	-	2.21 mg/dL	0.4-1.4 mg/dL

 

## Discussion

Erythrocytosis can be primary (polycythemia vera) or secondary, related to various disease conditions, drugs, and erythropoietin-producing malignancies. Secondary erythrocytosis is more common than the primary one [2]. SGLT2i are a recent addition to the list of drugs associated with secondary erythrocytosis. We observed two cases of dapagliflozin-associated erythrocytosis. Gupta et al. reported two cases from India, one related to canagliflozin and the other due to empagliflozin. Both were middle-aged diabetic males (59 and 46 years old). They observed a rise in Hb and hematocrit after two years of drug use. Both patients responded to drug discontinuation and therapeutic phlebotomy [4]. Contrastingly, both patients in our case had erythrocytosis within a short period of time. This could be due to the presence of CKD in both cases. Hemoconcentration, combined with erythrocytosis stimulation by dapagliflozin, could have contributed to the early development of secondary erythrocytosis in our patients.

In our cases, there was a good response to drug discontinuation, and Hb levels reduced substantially within one to two months of stopping the drug. A study by Gangat et al. observed 30 cases of SGLT2i-related erythrocytosis, of which 17, 9, and 4 cases were due to canagliflozin, empagliflozin, and dapagliflozin, respectively. Risk factors such as smoking and obstructive sleep apnea were seen in eight patients. The median level of peak Hb was 17.9 g/dL. Phlebotomy, antiplatelet therapy, and anticoagulation were given to 7, 14, and 5 cases, respectively. However, they discontinued SGLT2i in only seven cases, with only three cases due to erythrocytosis. Resolution of erythrocytosis was reported after a median of two months of drug discontinuation [5]. In understanding the mechanisms of erythrocytosis, hemoconcentration associated with SGLT2i may not be the sole reason. In combination, stimulation of erythrocytosis with erythropoietin production in native kidneys and action on HIFs contribute to it [3]. We did not analyze the erythropoietin levels in our cases. The response to treatment discontinuation highlights that both cases had SGLT2i-dapagliflozin-induced erythrocytosis. The renal function either improved or remained stable during and after the discontinuation of dapagliflozin [6,7].

## Conclusions

This is probably the first report of dapagliflozin-associated erythrocytosis in patients with T2DM and CKD. The occurrence of erythrocytosis within a short period (~6.5 months) of time probably indicates CKD patients may be at risk for this effect with the use of SGLT2i. We advise a routine evaluation of CKD patients treated by SGLT2i with a hemogram at regular intervals to detect and appropriately manage erythrocytosis to prevent the associated complications.
